# A forgotten double-J ureteral stent resulting in an emphysematous perinephric abscess: A case report

**DOI:** 10.1097/MD.0000000000029418

**Published:** 2022-06-24

**Authors:** In Hee Lee, Hong Seok Shin, Dong Jik Ahn

**Affiliations:** a Department of Internal Medicine, Daegu, Republic of Korea; b Department of Urology, Daegu Catholic University School of Medicine, Daegu, Republic of Korea; c Department of Internal Medicine, HANSUNG Union Internal Medicine Clinic and Dialysis Center, Daegu, Republic of Korea.

**Keywords:** double-J stent, perinephric abscess, urinary tract infection

## Abstract

**Rationale:**

Double-J stents (DJSs) are urologic devices widely used for urinary tract obstruction treatment. Perinephric abscess is a condition with purulent accumulation resulting from urinary tract infection retained between the renal capsule and Gerota's fascia. Emphysematous urinary tract infection in patients with a forgotten DJS is extremely rare. Herein, we report a case of emphysematous perinephric abscess as a complication in a 56-year-old non-diabetic woman who neglected a 10-year-old DJS placed for obstructive uropathy treatment.

**Patient concerns:**

The patient presented with fever and abdominal pain that persisted for 4 days. Laboratory examinations showed leukocytosis, hypoalbuminemia (2.3 g/dL), and elevated C-reactive protein level (305.5 mg/L) with no azotemia.

**Diagnosis:**

Abdominal computed tomography scan revealed a DJS with encrustation and multiple stones in the right kidney as well as a perinephric abscess with gas formation.

**Interventions:**

Intravenous administration of piperacillin/tazobactam was initiated immediately and percutaneous catheter drainage was performed. Extended-spectrum beta-lactamase-producing *Escherichia coli* was identified on abscess culture and antibiotics were switched to meropenem, resulting in gradual improvement of the inflammatory lesion. The patient was referred to the urology department for retained DJS removal and vesicolitholapaxy. A piece of fractured stent was removed via open ureterolithotomy.

**Outcomes:**

Since discharge on hospital day 42, she has been under regular follow-up, and the surgical wound has been healing with no significant sequelae.

**Lessons:**

Prompt medical therapy for inflammation and thorough urologic correction of the stent-induced structural deformities are crucial in long-term neglected DJS and resulting emphysematous perinephric abscess. Patients who undergo DJS placement should be systematically followed up to prevent potential neglect of device management.

## 1 Introduction

Double-J stents (DJSs), first introduced in 1967 by Zimskind et al, are devices widely used in the treatment of urologic diseases including ureteral obstruction and ureteral fistula, as well as in ureteral reconstruction.^[1]^ However, failure to remove the stent within 3 months may elevate the risk of unexpected complications, such as urinary tract infection (UTI), stent displacement, encrustation, stone formation, and stent fracture. In particular, prolonged retention of ureteral stents due to patient neglect or lack of medical awareness is a risk factor for severe complications, including structural alteration, inflammatory stone formation, hydronephrosis, and renal failure.^[2]^ Perinephric abscess is a condition in which UTI-induced perinephric fat necrosis leads to an accumulation of purulent secretions between the renal capsule and Gerota's fascia, resulting in high morbidity and mortality.^[3]^ The major antecedents of a perinephric abscess include UTI, stone formation, trauma, diabetes mellitus, pregnancy, immunosuppressed state, and structural anomaly of the urinary tract.^[4]^ Structural anomalies of the urinary tract include staghorn calculi, neurogenic bladder, severe vesicoureteral reflux, obstructive uropathy, papillary necrosis, renal cysts, and polycystic kidney disease.^[3]^ Cases of long-term neglected DJSs complicated by severe emphysematous UTI are extremely rare.^[5]^ Herein, we report a case of emphysematous perinephric abscess in a patient with a DJS forgotten for 10 years after stent placement for decompression of an upper ureteral obstruction. We also review the relevant literature.

## 2 Case presentation

A 56-year-old woman was transferred to our emergency department (ED) from a general hospital with chief complaints of fever and lower abdominal pain that had persisted for 4 days. She did not have a history of hypertension, diabetes mellitus, tuberculosis, chronic liver, or cardiac diseases. She had undergone DJS placement 10 years earlier at a different hospital due to ureteral stricture with UTI. A peripheral blood test performed 2 days before presentation to our institute yielded the following results: white blood cell (WBC) count, 13,390/μL; hemoglobin level, 7.2 g/dL; platelet count, 556,000/μL; and C-reactive protein (CRP) level, 303 mg/L (reference range, <5 mg/L). Initially, a complicated UTI was suspected, and intravenous (IV) administration of antibiotics (ceftriaxone and netilmicin) and transfusion of packed red blood cells were performed. However, high fever, nausea, vomiting, dysuria, and urinary frequency persisted, resulting in transfer to our tertiary hospital.

**Figure 1:**
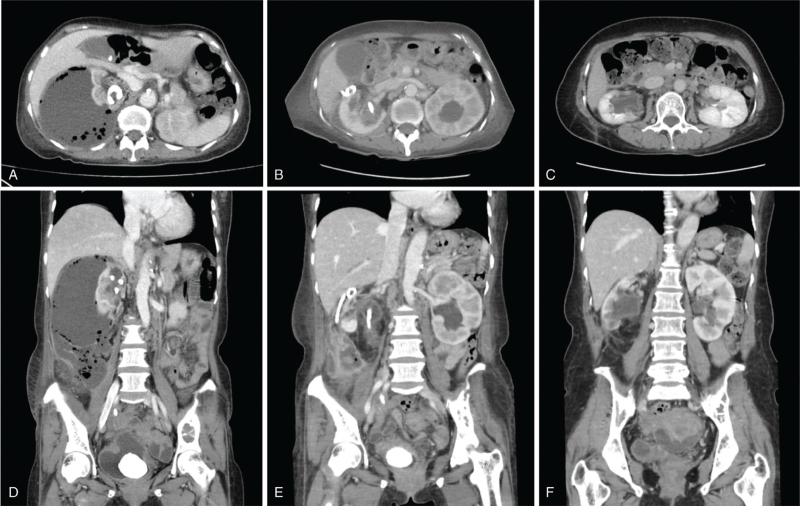
(A, D) Abdominal computed tomography (CT) scans on admission demonstrated a large perinephric abscess with gas formation and a forgotten double-J stent (DJS) in the right kidney. (B, E) Abdominal CT scans on hospital day 8 showed marked improvement of the right perinephric abscess after percutaneous catheter drainage. (C, F) Follow up abdominal CT two months after open ureterolithotomy revealed no significant inflammatory lesions. CT = computed tomography, DJS = double-J stent.

**Figure 2:**
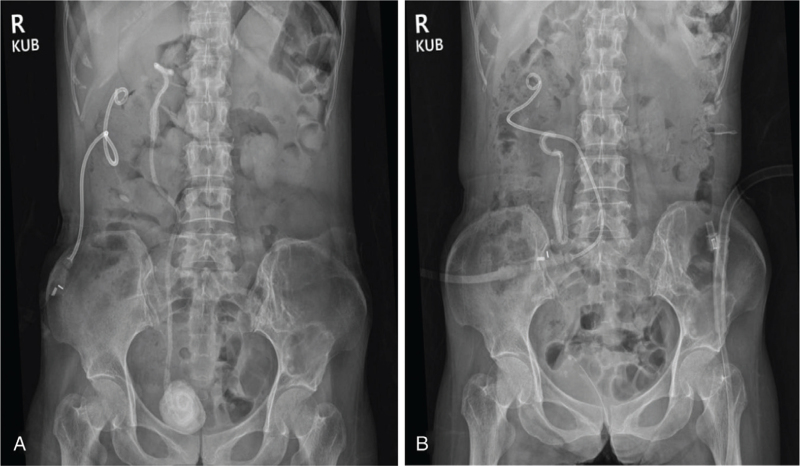
(A) X-ray Kidney-ureter-bladder (KUB) after percutaneous catheter drainage of right perinephric abscess. This revealed a double-J stent (DJS) with a large bladder stone formed in the lower coil. (B) On the 12th hospital day, a KUB scout image showed a DJS remnant adhered to the right proximal ureter and renal pelvis after endoscopic vesicolitholapaxy. DJS = double-J stent, KUB = kidney-ureter-bladder.

On arrival at our ED, the patient had a blood pressure of 136/83 mm Hg, pulse rate of 110/min, respiration rate of 20/min, and body temperature of 38.3 °C. She was alert but appeared chronically ill. A physical examination revealed that the lower abdomen was mildly distended without rebound tenderness. However, right costovertebral angle tenderness was observed. A peripheral blood test yielded the following results: WBC count, 13,400/μL (neutrophils, 81.9%); hemoglobin level, 9.3 g/dL; platelet count, 487,000/μL; and erythrocyte sedimentation rate, 96 mm/h. The serum biochemistry profile was as follows: albumin, 2.3 g/dL; blood urea nitrogen, 25.1 mg/dL; creatinine, 0.6 mg/dL; and CRP, 305.5 mg/L. Routine urinalysis showed the following results: albumin, 1+; leukocyte esterase, 3+; and nitrite, negative. Microscopic examination of the urinary sediment revealed WBCs > 30/high power field and red blood cells > 30/high power field. Serologic tests for hepatitis B surface antigen, anti-hepatitis C virus antibody, anti-human immunodeficiency virus antibody, anti-nuclear antibody, and severe acute respiratory syndrome coronavirus 2 polymerase chain reaction test were all negative. However, abdominal computed tomography (CT) scan with contrast enhancement indicated a huge perinephric abscess with abundant gas formation in the right kidney (Fig. 1A and D). Broad-spectrum IV antibiotics (piperacillin/tazobactam 4.5 g, ×2/d) and metronidazole (500 mg, ×4/d) were prescribed. In addition, image-guided percutaneous catheter drainage (PCD) for right perinephric abscess was performed (Fig. 2A). Over the following three days, a copious amount of suppurative exudates (>3 L/d) via PCD were drained. On hospital day (HD) 4, extended-spectrum beta-lactamase-producing *Escherichia coli* (*E coli*) was detected in cultures of the drained abscess and voided urine. However, blood cultures were negative for bacteria. Considering the persistent fever, laboratory data (e.g., elevated WBC count and CRP level), and antibiotics susceptibility test results since hospital admission, the antibiotics were switched to meropenem (1.0 g, ×3/d).

**Figure 3:**
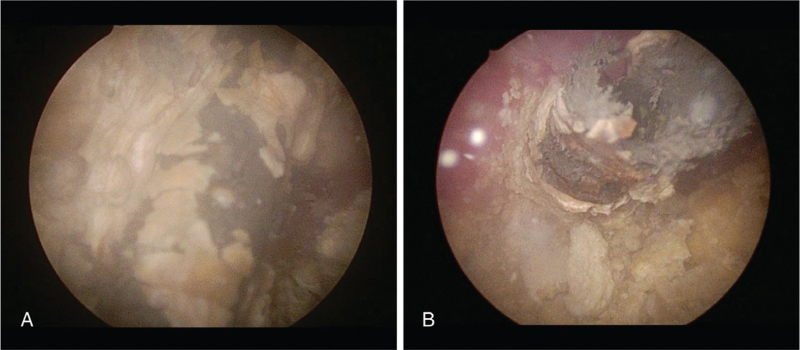
(A) Endoscopic view of a double-J stent (DJS) surrounded with extensive encrustation. (B) Endoscopic view of right ureterovesical junction showing multiple small stones around DJS. DJS = double-J stent.

**Figure 4:**
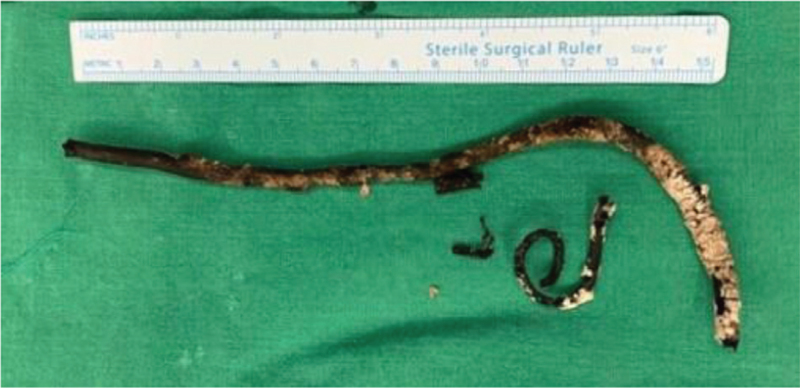
A forgotten double-J stent extracted from the urinary bladder. Encrusted stent was fractured and only two-thirds of the distal part was removed.

On HD 8, follow-up abdominal CT showed a marked improvement of the inflammatory lesions in the right kidney. However, a large bladder stone formed in the lower coil of DJS led to obstructive hydronephrosis in the left kidney (Fig. 1B and E). On HD 12, the patient underwent endoscopic vesicolitholapaxy with ureteral stones removal (Fig. 3). A 3.9-cm stone within the urinary bladder was crushed using laser lithotripsy and removed. However, the DJS was fractured, and only two-thirds of the distal part was removed (Fig. 4). The proximal portion of the DJS had adhered to the right renal pelvis (Fig. 2B). On HD 17, percutaneous nephrostomy was performed to achieve secondary decompression in the right kidney. Removal of the renal stones and DJS remnant was attempted along with antegrade stent placement, but this procedure failed. Therefore, while continuing supportive therapy, an open ureterolithotomy was implemented on HD 32 to completely remove the renal stones and encrusted DJS remnant. On HD 42, the patient showed improvement in clinical symptoms and laboratory findings, including WBC count, 8,900/μL; creatinine level, 0.4 mg/dL; and CRP level, 12.8 mg/L, and she was discharged. Since discharge from the hospital, she has been under regular follow-up, and the surgical wound has been healing without significant sequelae. Follow up abdominal CT two months after open ureterolithotomy revealed no specific complications (Fig. 1C and F).

## 3 Discussion

DJSs or pigtail stents are indwelling urologic devices widely used to alleviate ureteral edema and ensure proper ureteral patency in patients with obstructive urinary lesions induced by intrinsic or extrinsic factors.^[1]^ Stent placement is highly useful and efficient for the postoperative management of patients with ureteral stones, ureteral stricture, retroperitoneal fibrosis or tumors, ureteropelvic obstruction, and any type of iatrogenic ureteral injury. ^[2]^ However, indwelling DJSs may lead to diverse and unexpected complications. Short-term complications include infection, hematuria, pain, and stent syndrome; however, if a stent is retained for 12 weeks or longer, stone formation, encrustations, stent fracture or blockage, hydronephrosis, renal dysfunction (occasionally), and recurrent UTI may occur.^[6]^ The incidence of encrustations over a stent surface increases proportionally with the duration of stent indwelling and is associated with urine composition, infection status, and metabolic or congenital anomalies. A history of urinary stones, active infection, and stent material are also major factors related to encrustations.^[7]^ Based on these potential complications following stent placement, ureteral stents should be replaced or removed within 6 weeks to 6 months.^[8]^ If a ureteral stent is forgotten for more than a year, extracorporeal shock wave lithotripsy, ureteroscopy, percutaneous nephrolithotomy, and/or open surgery may be required to remove the stent.^[7,9]^

In the present case, the stent was fractured during the first removal attempt, and two more rounds of percutaneous nephrostomy were unsuccessful. Eventually, an open urologic surgery was required. While stents can fracture spontaneously, breakage of the stent during the removal procedure was due to the severely encrusted tips and tight adhesion of the upper part of the stent to the renal pelvis. In addition, the possibility of stent hardening and loss of tensile strength cannot be excluded. ^[2]^ In a study on 30 patients with DJSs retained for 6 months or longer, the most common symptom at the time of presentation was dysuria (24/30, 80%), followed by lower urinary tract symptoms (53.3%), hematuria (40%), and flank pain (30%). However, recurrent UTI was less common (26.7%).^[10]^ Our patient seems to have overlooked the persistence of these nonspecific urinary symptoms and lacked medical awareness about DJS management. Thus, patients with an indwelling ureteral stent should be closely followed for systematic stent management and potential stent-related urinary complications in consideration of their socioeconomic status, education level, and compliance with urologic intervention.

Perinephric abscess triggers a progressive manifestation of nonspecific general symptoms or signs, such as high fever, chill, flank pain, general weakness, lethargy, and weight loss over several weeks, and these may delay the diagnosis.^[3,11]^ Fever and chill are the most common symptoms, while the typical symptoms of UTI, namely dysuria and urinary frequency, are relatively less frequent.^[3,12]^ Many previous reports have documented hematogenous dissemination of suppurative lesions caused by other staphylococcal infections, such as wound infection, furuncles, and pulmonary infection resulting in renal abscesses, which might rupture and drain to other spaces leading to secondary perinephric abscesses.^[13]^ In recent years, however, the number of perinephric abscess cases related to ascending UTI from Gram-negative enteric bacteria, particularly *E coli*, *Klebsiella pneumoniae*, and *Proteus* spp., and fungal infections, including *Candida* spp. infection, have been increasing.^[14]^ Emphysematous perinephric abscess is caused by an extravasation of purulent substances in the renal parenchyma generated by gas-forming bacteria, such as *E coli*, *Klebsiella* spp., and *Pseudomonas* spp., into the perinephric space owing to elevated intrapelvic pressure.^[15]^

Emphysematous perinephric abscess is also considered a progressive disease in the same spectrum of emphysematous pyelonephritis characterized by intrarenal, or at times, perinephric gas formation.^[11,16]^ However, the abdominal CT findings of our patient indicated that inflammatory gas formation was virtually confined to the perirenal area (not within the renal parenchyma), and this caused external compression to the renal parenchyma strongly suggesting the presence of an emphysematous perinephric abscess rather than emphysematous pyelonephritis.^[16]^

The recommended first-line treatment for a large perinephric abscess (>3 cm) includes the use of antimicrobial agents with PCD, because the abscess may expand into other adjacent retroperitoneal organs and the perinephric space.^[4]^ However, if medical treatment fails, surgical interventions should be actively considered to resolve the abscess.^[3,4]^ A recent report described the case of a patient with a forgotten stent complicated by emphysematous pyelonephritis and septic shock sequelae despite receiving appropriate antibiotic therapy and percutaneous drainage who ultimately underwent nephrectomy.^[5]^ Our patient presented with fever and abdominal pain and showed urosepsis with acute obstructive pyelonephritis. Upon arrival at the ED, she was immediately given IV administration of broad-spectrum antibiotics, fluid therapy, and PCD. Fortunately, the perinephric abscess improved rapidly, and on HD 8, the patient was referred to the urology department for radical correction of the stent-related structural abnormalities. We removed the encrusted stent via an endourological approach and performed cystolithotomy for bladder stone removal. Although an open surgery was inevitable to remove the fractured stent remnant, the patient was able to recover completely without nephrectomy. We speculate that the patient showed a relatively favorable outcome despite having a huge abscess with much emphysema because she had no poor prognostic factors of perinephric abscesses, such as old age, diabetes mellitus, trauma, renal insufficiency, altered consciousness, and immunodeficiency.^[12]^

In summary, we report a case of long-term neglected DJS complicated by an emphysematous perinephric abscess and bladder stones. Our case findings suggest that while patients with long-forgotten DJSs are rare, such patients can develop severe perinephric abscesses and require prompt medical treatment for the inflammatory lesion and thorough urologic correction of the stent-induced structural deformities. Our findings also highlight the importance of systematic follow-up of patients with indwelling DJSs to prevent potential neglect of these stents for prolonged periods.

## Author contributions

**Conceptualization:** In Hee Lee.

**Data curation:** In Hee Lee, Hong Seok Shin.

**Formal analysis:** In Hee Lee, Dong Jik Ahn.

**Methodology:** In Hee Lee, Hong Seok Shin.

**Validation:** Hong Seok Shin, Dong Jik Ahn.

**Writing – original draft:** In Hee Lee.

**Writing – review & editing:** In Hee Lee.
